# Effectiveness of Manual Lymphatic Drainage After Total Knee Arthroplasty: A Systematic Review

**DOI:** 10.3390/jcm15145575

**Published:** 2026-07-16

**Authors:** Papi Davide, Carulli Christian, Lorenzoni Niccolo’, Montigiani Giulia

**Affiliations:** 1 Careggi University Hospital, University of Florence, 50134 Florence, Italy; davide.papi@unifi.it (P.D.); niccolo.lorenzoni@edu.unifi.it (L.N.); montigianigi@aou-careggi.toscana.it (M.G.); 2 Orthopaedic Clinic, University of Florence, 50134 Florence, Italy

**Keywords:** edema, total knee arthroplasty, manual lymphatic drainage

## Abstract

**Background/Objectives**: Total Knee Arthroplasty (TKA) is the gold-standard treatment for end-stage knee osteoarthritis. After surgery, the natural onset of edema and pain usually influences the early functional recovery. Manual Lymphatic Drainage (MLD) has been proposed as a possible adjunct rehabilitation strategy in the immediate management of the operated joint. This review aimed to assess the effectiveness of MLD during early rehabilitation after TKA. **Methods**: Searches were conducted in PubMed, Embase, CINAHL, and Google Scholar up to June 2025. Eligible studies were randomized controlled trials (RCTs) comparing MLD with non-MLD interventions (standard physiotherapy, Kinesiotaping, and negative-pressure devices). An exploratory meta-analysis using a random-effects model was performed. The primary outcome was postoperative edema reduction; secondary outcomes were the effects on pain and range of motion (ROM). **Results**: Eight RCTs (n = 463 participants) met the inclusion criteria. Edema data were insufficient for reliable quantitative synthesis (two poolable studies; SMD = −1.08 and +0.20; I^2^ = 78.1%). MLD did not yield consistent improvements in AROM (SMD = 0.15; 95% CI: −0.08 to 0.37; I^2^ = 0.0%) or pain. Two studies reported modest, short-lived benefits in the first postoperative days. No treatment-related adverse events were reported. **Conclusions**: Available evidence does not support the routine use of MLD as a primary intervention after TKA. Owing to its excellent safety profile, MLD may be considered selectively within a multimodal rehabilitation program as an adjunctive comfort-enhancing intervention, particularly for patients with low exercise tolerance. Further high-quality RCTs with standardized protocols are needed.

## 1. Introduction

Osteoarthritis (OA) is a chronic inflammatory joint disease characterized by progressive degeneration of articular cartilage, subsequently extending to subchondral bone and surrounding synovial structures. Among the most common affected joints, knee accounts for approximately 85% of OA cases worldwide [1,2,3,4]. The principal clinical manifestations—pain, reduced mobility, and swelling—progressively impair functional capacity. Total Knee Arthroplasty (TKA) is currently considered the most effective treatment for end-stage knee OA. According to the 2024 Annual Report of the American Joint Replacement Registry, TKA accounted for 51.1% of 3,715,320 arthroplasty procedures performed between 2012 and 2023 [5]. The number of TKAs is expected to increase by approximately 673% in the United States by 2030 [6].

Despite the proven clinical efficacy of TKA, approximately 20% of patients report low satisfaction one year after surgery, primarily due to persistent pain and limited functional improvements [7]. Several factors may contribute to this degree of suboptimal outcome: early postoperative complications including persistent edema and pain, joint stiffness, infection, instability, deep vein thrombosis, underestimated neuropathy related to lumbar spine issues, hypersensitivity to metals or cement, and even an unjustified indication for TKA at the time of surgery [8,9,10]. Among these, edema and pain are particularly prevalent and can significantly delay functional recovery [11,12,13,14,15,16,17,18,19].

Postoperative edema following TKA is a natural and expected lymphodynamic soft tissue swelling—excess interstitial fluid resulting from surgical trauma. It follows a three-phase evolution: the inflammatory phase (POD 3–5), the fibroblastic phase (week 2 to approximately week 6), and the remodeling phase (month 6 to up to 2 years) [12]. Appropriate phase-specific management is essential to prevent progression to fibrotic transformation [13]. Pain is also naturally present after major surgical procedures such as TKA, despite various pharmacological and non-pharmacological aids [14,15,16,17].

Manual Lymphatic Drainage (MLD) is a gentle, superficial manual technique developed in the 1960s by Emil Vodder. It targets superficial lymphatic vessels through rhythmic pumping maneuvers aimed at stimulating lymphangiomotor activity and promoting lymphatic transport and edema reduction [18,19,20,21,22]. MLD constitutes one component of Complete Decongestive Therapy (CDT), alongside compression bandaging, skin care, and therapeutic exercise. Despite its role in lymphedema management, evidence for its use in post-TKA rehabilitation remains inconclusive, despite two recent systematic reviews published on this topic [23,24].

The present systematic review aimed to critically evaluate the available evidence from RCTs regarding the effectiveness of MLD on edema, pain, and ROM in the early postoperative period following TKA, and to provide recommendations for clinical practice and future research.

## 2. Materials and Methods

### 2.1. Study Design

This study was conducted as a systematic review of RCTs. Reporting follows the Preferred Reporting Items for Systematic Reviews and Meta-Analyses (Supplementary Materials PRISMA 2020 Checklist) [25]. A review protocol was developed by the authors; however, it was not prospectively registered in PROSPERO. The protocol defined postoperative edema reduction as the primary outcome, assessed within the early postoperative period (up to 6 weeks after surgery). Secondary outcomes included pain intensity and Active Range Of Motion (AROM). Eligible study designs were limited to RCTs comparing MLD with any non-MLD comparator in adult patients undergoing primary TKA.

### 2.2. Eligibility Criteria

Studies were considered eligible according to the following PICO framework (Table 1):

Exclusion criteria: reviews, meta-analyses, conference proceedings, case reports, duplicate publications, studies with overlapping data, ambiguous methodology, incomplete outcome data, and articles published in languages other than English. Studies were considered eligible only if MLD was explicitly defined as a gentle manual massage technique performed by trained therapists along specific lymphatic pathways, or if the authors reported adherence to the Vodder or Földi method; this definition was applied a priori during title, abstract, and full-text screening.

### 2.3. Information Sources and Search Strategy

A systematic literature search was conducted in PubMed (MEDLINE), Embase, CINAHL, and Google Scholar up to June 2025. Search queries were developed using MeSH terms and free-text keywords for ‘Manual Lymphatic Drainage,’ ‘Arthroplasty, Replacement, Knee,’ and ‘Edema.’ Complete search strings are provided in Supplementary Materials. No time restrictions were applied. Search terms were tailored to each database in order to account for interchangeable use of related terminology across the literature, and the PubMed search was additionally sorted using the platform’s relevance-ranked ‘Best Match’ algorithm. Google Scholar was used exclusively for citation chasing (identifying additional references from the bibliography of included studies and relevant systematic reviews) and was not used as a primary screening database.

### 2.4. Study Selection and Data Extraction

Titles and abstracts screening followed by full-text eligibility assessment was performed by a single reviewer. Reference lists of included studies and relevant systematic reviews were manually screened to minimize omissions. Study management was performed using Mendeley software (2.141.0 version). Full texts of potentially eligible articles were retrieved through institutional subscription access to the identified databases and journals.

Data extracted for each study included: first author, year of publication, country, total and per-group sample size, mean age, intervention details (technique, session duration, frequency, number of sessions, timing), comparator, co-interventions, and outcome measurements at each time point. For studies with more than two intervention arms, the number and composition of comparison groups were additionally recorded to guide subsequent data synthesis.

### 2.5. Methodological Quality Assessment

The methodological quality of included studies was assessed using the Cochrane Risk of Bias 2 (RoB 2) tool for RCTs, examining five domains: randomization process, deviations from intended interventions, missing outcome data, outcome measurement, and selection of reported results.

### 2.6. Statistical Analysis

An exploratory meta-analysis was conducted for outcomes with a minimum of two comparable studies. A random-effects model (restricted maximum likelihood [REML] estimator) was used to pool effect sizes as Standardized Mean Differences (SMD), with Knapp–Hartung adjustments applied to confidence intervals. Heterogeneity was quantified using the I^2^ statistic. All analyses were performed in R^®^ (Rstudio, 2025.09 version). Outcomes not amenable to meta-analysis were reported narratively.

## 3. Results

**Figure 1:**
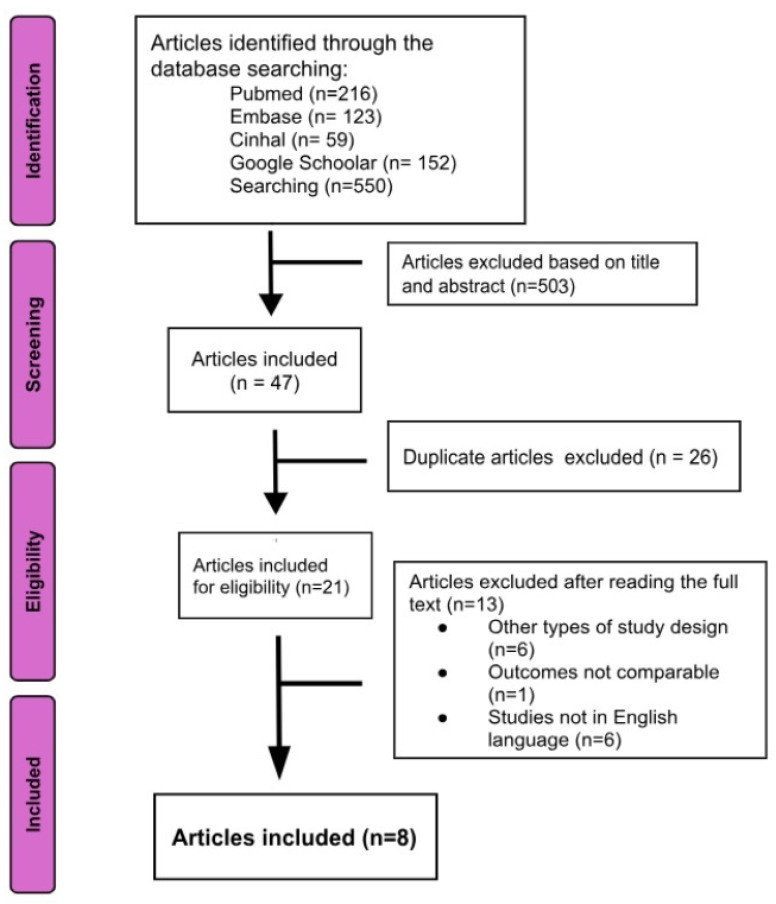
PRISMA flow diagram.

Database searches yielded 550 records. Following title and abstract screening, 47 articles were retrieved; 26 duplicates were removed. After full-text evaluation, 13 articles were excluded. Eight RCTs were ultimately included in the systematic review (Figure 1).

The eight included RCTs enrolled a total of 463 participants who had undergone primary TKA. Studies were conducted in Italy, Switzerland, Turkey, Australia, Japan, Austria, and Germany, published between 2013 and 2025. Patient mean age ranged from 63.5 to 71.3 years (Table 2).

Regarding interventions, five studies compared MLD (plus standard physiotherapy) versus standard physiotherapy or placebo; two three-arm studies compared MLD versus Kinesiotaping (KT) versus standard care; one study compared timing of MLD delivery (pre- and postoperative versus postoperative-only); and one study compared MLD with a negative-pressure device. Session duration ranged from 20 to 30 min per day, with 2–10 total sessions (Table 3).

### 3.1. Outcomes and Follow-Up

Edema was the prespecified primary outcome across all included studies. It was measured via limb circumferences at various anatomical landmarks (five studies), volumetric methods (three studies), or bioelectrical impedance spectroscopy (one study). Pain was assessed with a Visual Analog Scale (VAS) or Numeric Rating Scale (NRS); Active Range of Motion (AROM) and passive Range of Motion (PROM) were measured with a manual goniometer. Follow-up ranged from POD 6 [26] to 3 months [27]. Baseline assessments were performed preoperatively in six of eight studies.

### 3.2. Risk of Bias

**Figure 2:**
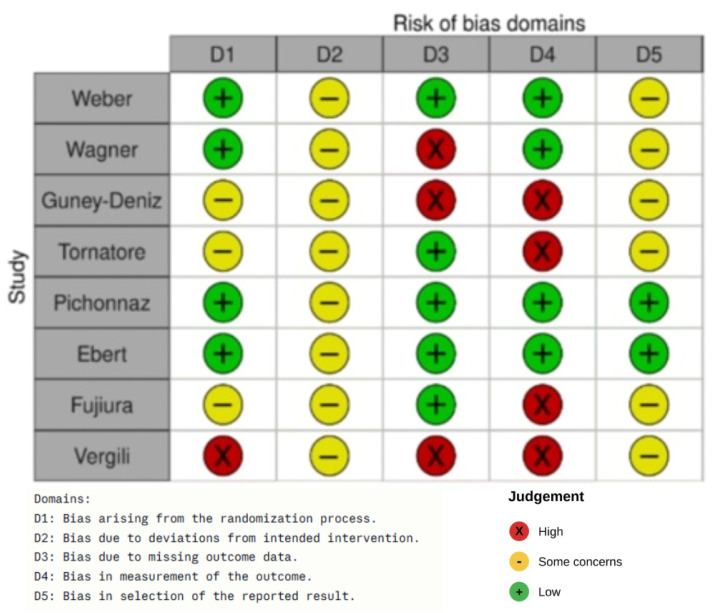
Risk of bias summary for included studies.

Assessment with the Cochrane RoB 2 tool revealed variable methodological quality. Blinding of participants and personnel was not feasible given the nature of the intervention, introducing a high or unclear risk of bias for the ‘deviations from intended interventions’ domain in most studies. Randomization and outcome measurement were generally adequate. Only one study enrolled more than 100 participants; most were underpowered (Figure 2).

### 3.3. Meta-Analysis Results

1. Effect on Edema

**Figure 3:**
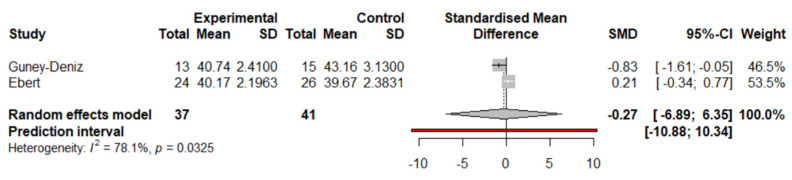
Forest plot of the incidence of MLD on edema.

Of eight included studies, only two (Guney-Deniz et al. [30] and Ebert et al. [32]) provided edema data amenable to pooling. The remaining six were excluded due to: heterogeneous measurement methods and anatomical landmarks (circumferences at varying sites, volumetry, bioelectrical impedance spectroscopy); multi-arm designs with mixed comparators (Tornatore et al. [31], Wagner et al. [29]); and one study comparing MLD against a negative-pressure device rather than standard care (Weber et al.) [28]. A formal meta-analysis was attempted but the resulting estimate cannot be interpreted reliably. The two studies yielded effect sizes in opposite directions (SMD = −1.08 and SMD = +0.20, respectively), producing an I^2^ of 78.1%; a level of heterogeneity that, with only two studies, is statistically unstable and precludes meaningful pooling. Accordingly, these data are better interpreted as insufficient for quantitative synthesis. Narratively, neither study demonstrated a statistically significant between-group difference in edema reduction (Figure 3).

2. Effect on Active Range of Motion

**Figure 4:**
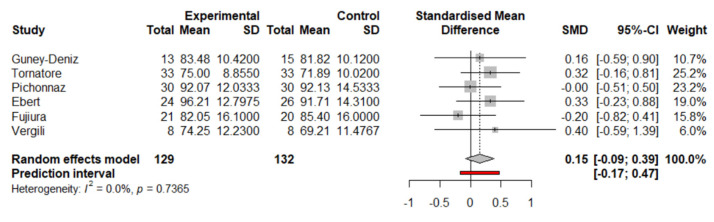
Forest plot of the incidence of MLD on AROM.

Six studies contributed data on active knee flexion AROM. The pooled SMD was 0.15 (95% CI: −0.08 to 0.37; *p* = 0.19), with no significant heterogeneity (I^2^ = 0.0%; *p* = 0.7365). MLD did not significantly improve AROM compared with control interventions. This finding reflects the limits of the available data rather than a definitive conclusion of clinical ineffectiveness (Figure 4).

### 3.4. Narrative Synthesis of Individual Study Results

Pichonnaz et al. (2016): In 56 patients (MLD + standard physiotherapy vs. placebo + standard physiotherapy; 5 sessions, POD 2–7, 30 min/day), no significant between-group differences were found for edema, pain, or ROM at any time point, except for lower passive knee flexion contracture in the MLD group at 3 months. An immediate but transient post-session analgesic effect was observed [27].

Wagner et al. (2024): In 112 patients randomized to postoperative-only MLD, pre- and postoperative MLD, or standard physiotherapy (5 sessions, 30 min/day), no statistically significant differences were found for WOMAC, pain, ROM, or edema at any time point. Preoperative MLD conferred no additional benefit [29].

Ebert et al. (2013): In 50 patients (MLD + standard physiotherapy on POD 2–4 vs. standard physiotherapy alone, 30 min/day), the MLD group showed greater active knee flexion at 6 weeks, while edema, pain, and KOOS did not differ significantly between groups [32].

Fujiura et al. (2020): In 40 patients (20 min MLD before standard physiotherapy until POD 10 vs. standard physiotherapy alone), no significant differences were found for pain, edema, ROM, or gait parameters [33].

Guney-Deniz et al. (2023): In 40 patients (MLD + standard physiotherapy vs. KT + standard physiotherapy vs. standard physiotherapy alone), both MLD and KT groups demonstrated less thigh/calf edema and pain than controls at POD 4 and at 2 weeks. No between-group differences persisted at 6 weeks [30].

Tornatore et al. (2020): In 99 patients (MLD + KT vs. MLD alone vs. KT alone), the MLD + KT combination reduced edema and pain more than either treatment alone in the acute phase. No between-group ROM differences were detected [31].

Vergili et al. (2022): In 16 patients (MLD + standard physiotherapy on POD 2 and POD 4 vs. standard physiotherapy alone), no significant differences were found for edema, pain, ROM, or FIM scores [26].

Weber et al. (2025): In 50 patients (MLD 20 min/day POD 1–7 vs. negative-pressure therapy with LymphaTouch), both interventions demonstrated equivalent effectiveness for edema reduction and ROM improvement. Negative-pressure therapy showed superior pain reduction on POD 2 and POD 4 [28].

## 4. Discussion

This systematic review synthesized data from eight RCTs evaluating MLD in early post-TKA rehabilitation. MLD did not produce statistically significant reductions in postoperative edema compared with control interventions. Whether this reflects a true absence of clinical benefit or insufficient statistical power to detect a meaningful effect remains uncertain, given the consistently small sample sizes across included studies.

These findings are consistent with the broader literature on MLD [23,24]. Gilchrist et al. (2024) [34] reported limited and uncertain evidence for MLD in limb-volume reduction. Conversely, Ezzo et al. (2015) [35] demonstrated that MLD combined with multilayer compression bandaging is effective in early-stage secondary lymphedema after breast cancer treatment, and Thompson et al. (2021) [36] identified possible beneficial effects on volume and quality of life, though without additional benefit in moderate-to-severe lymphedema. The post-TKA edema model differs importantly from breast cancer-related lymphedema—it involves transiently overloaded but structurally intact lymphatics—which may explain the limited MLD response. This distinction may be further explained mechanistically: according to Leduc, MLD is effective only when functional lymphatic vessels remain available for stimulation within the treated area, and is rarely sufficient as a stand-alone treatment for edema. Vodder likewise observed that not all interstitial fluid is drainable through manual stimulation alone, since the protein-rich component of more organized edema is comparatively resistant to mobilization by MLD.

Several methodological limitations constrain the available evidence. Sample sizes were generally small (range 16–112; only one study exceeded 100 participants), reducing statistical power. Follow-up was predominantly short (within the first two weeks), with only two studies extending to 6 weeks or beyond. Edema measurement relied heavily on limb circumferences, a tool with limited sensitivity for small intraday changes. Furthermore, MLD protocols were poorly standardized across studies—varying in technique (Vodder vs. Földi), session duration (20–30 min), frequency, total number of sessions, and the pressure applied—making it impossible to define an optimal therapeutic window or dose–response relationship.

Two findings deserve further consideration. First, the transient benefits reported by Guney-Deniz et al. [30] and Tornatore et al. [31] in the acute phase suggest that MLD may provide short-term symptomatic relief that, even if not sustained, could enhance patient comfort and adherence to early rehabilitation. Second, across all eight studies, MLD was not associated with any adverse events, confirming its excellent safety profile in this population. Additionally, synthesis of individual study data offers further nuance: two of the eight trials (Pichonnaz et al. [27] and Ebert et al. [32]) reported a modest benefit of MLD on active knee flexion, and Pichonnaz et al. [27] also observed an immediate, though transient, analgesic effect after most treatment sessions; conversely, none of the included studies demonstrated a statistically significant reduction in edema with MLD.

This review has some limitations. The protocol was not prospectively registered in PROSPERO, which may reduce transparency and increase the risk of methodological deviations during the review process. Furthermore, publication bias could not be formally assessed. The small number of included studies, and particularly the limited number of studies contributing to each meta-analysis, precluded reliable evaluation through funnel plots or statistical tests. Consequently, the possibility that unpublished studies with negative findings exist cannot be excluded. Finally, the study selection and eligibility assessment were performed by a single reviewer rather than by two independent reviewers, and no duplicate data extraction was performed. Although predefined eligibility criteria were applied, this approach may have increased the risk of selection bias and reduced the reproducibility of the review process.

Although a formal GRADE assessment was not performed, a qualitative appraisal of the certainty of evidence was conducted for each primary outcome. For edema, certainty was rated very low, given the insufficient number of poolable studies, high heterogeneity, directionally inconsistent effect sizes, and serious methodological concerns. For pain, certainty was rated low, due to variable measurement tools, small sample sizes, and inconsistent results across time points. For AROM, certainty was rated low-to-moderate: heterogeneity was minimal (I^2^ = 0.0%) and six studies contributed data, though sample sizes remained small and follow-up short. Overall, the current evidence is insufficient to draw definitive conclusions about the effectiveness of MLD after TKA. Based on these considerations, MLD should not be recommended as a routine, primary, standalone intervention for post-TKA edema management. However, the technique may have a role as an adjunctive intervention within a multimodal rehabilitation program, particularly for patients with low exercise tolerance or pain-related movement avoidance. Its use should be guided by clinical judgement and patient preference. Joined together, the available evidence suggests that MLD may represent a complementary option in the early phase of rehabilitation after TKA, particularly useful for short-term pain relief and for facilitating early mobilization, rather than an essential or independently sufficient component of the rehabilitation protocol.

## 5. Conclusions

This systematic review found no clear clinical superiority of MLD over comparator interventions in reducing postoperative edema, improving pain, or enhancing ROM after TKA. Edema data were insufficient for reliable quantitative synthesis, with only two considerable studies yielding heterogeneous and directionally inconsistent results, while the pooled meta-analysis confirmed the absence of statistically significant effects on AROM (SMD = 0.15; I^2^ = 0.0%). Current evidence is insufficient to confirm or exclude a clinically meaningful effect, and these findings should be interpreted in the context of the methodological limitations of the included studies, particularly their limited statistical power.

Given its excellent safety profile and the transient symptomatic benefits noted in some studies, MLD may be considered as an adjunctive intervention within a multimodal rehabilitation protocol for selected patients. Future well-powered RCTs with standardized MLD protocols, sensitive edema measurement tools (volumetry, bioelectrical impedance spectroscopy), and longer follow-up periods are needed to fully delineate the role of MLD after TKA.

## Figures and Tables

**Table 1:** PICO eligibility framework.

Parameter	Criteria
Population	Adult patients undergoing primary TKA
Intervention	MLD performed by trained therapists following the Vodder or Földi method, as the primary intervention
Comparison	Placebo, standard physiotherapy, Kinesiotaping (KT), or negative-pressure devices
Outcomes	Primary: postoperative edema (limb circumference, volumetry, BIS). Secondary: pain (VAS/NRS) and range of motion (ROM)
Study design	Randomized controlled trials, published in English, without year restrictions

**Table 2:** General characteristics of included studies.

Author	Year	Country	N (Total/Per Group)	Mean Age (Years)
Vergili	2022 [26]	Turkey	16 (8/8)	63.5 ± 9.3
Pichonnaz	2016 [27]	Switzerland	56 (29/27)	70.7 ± 8.0
Weber	2025 [28]	Germany	50 (25/25)	67.6
Wagner	2024 [29]	Austria	112 (36/37/39)	69.4 ± 9.8
Guney-Deniz	2022 [30]	Turkey	40 (13/12/15)	65.6 ± 3.5
Tornatore	2020 [31]	Italy	99 (33/33/33)	71.3 ± 6.8
Ebert	2013 [32]	Australia	50 (24/26)	70.0
Fujiura	2020 [33]	Japan	40 (20/20)	71.0

**Table 3:** Interventions across included studies.

Author	Group 1 (MLD)	Group 2	Control
Weber	MLD + FKT (20 min/day, POD 1–7)	—	NP device (LymphaTouch, 20 min/day)
Wagner	MLD post + FKT (30 min/day, POD 1–5)	MLD pre + post + FKT	FKT alone
Guney-Deniz	MLD + FKT (30 min/day, POD 2–4)	KT + FKT	FKT alone
Tornatore	MLD + KT (30 + 15 min, POD 2 & 4)	MLD alone	KT alone
Pichonnaz	MLD + FKT (30 min/day, POD 2–7)	—	Placebo + FKT
Ebert	MLD + FKT (30 min/day, POD 2–4)	—	FKT alone
Fujiura	MLD + FKT (20 min/day, up to POD 10)	—	FKT alone
Vergili	MLD + FKT (30 min, POD 2 & 4)	—	FKT alone

MLD: Manual Lymphatic Drainage; FKT: standard physiotherapy; KT: Kinesiotaping; NP: negative pressure; Post Operative Day (POD).

## Data Availability

No new data were created or analysed in this study. Data sharing is not applicable to this article.

## Supplementary Materials

The following supporting information can be downloaded at: https://www.mdpi.com/article/10.3390/jcm15145575/s1, PRISMA 2020 Checklist; Figure S1: Risk of bias summary for included studies; File S1: Search strings on: Pubmed, Embase, Cinhal, google scholar.

## References

[1] Steinhaus M.E., Christ A.B., Cross M.B. (2017). Total Knee Arthroplasty for Knee Osteoarthritis: Support for a Foregone Conclusion?. HSS J. Musculoskelet. J. Hosp. Spec. Surg..

[2] Giorgino R., Albano D., Fusco S., Peretti G.M., Mangiavini L., Messina C. (2023). Knee Osteoarthritis: Epidemiology, Pathogenesis, and Mesenchymal Stem Cells: What Else Is New? An Update. Int. J. Mol. Sci..

[3] Allen K.D., Thoma L.M., Golightly Y.M. (2022). Epidemiology of osteoarthritis. Osteoarthr. Cartil..

[4] Hunter D.J., Bierma-Zeinstra S. (2019). Osteoarthritis. Lancet.

[5] Du X., Liu Z.Y., Tao X.X. (2023). Research Progress on the Pathogenesis of Knee Osteoarthritis. Orthop. Surg..

[6] Carender C.N., Hegde V., Levine B.R., Huddleston J.I., Cohen-Rosenblum A. (2025). Highlights of the 2024 American Joint Replacement Registry Annual Report. Arthroplast. Today.

[7] Bourne R.B., Chesworth B.M., Davis A.M., Mahomed N.N., Charron K.D.J. (2010). Patient Satisfaction after Total Knee Arthroplasty: Who is Satisfied and Who is Not?. Clin. Orthop. Relat. Res..

[8] Carulli C., Villano M., Bucciarelli G., Martini C., Innocenti M. (2011). Painful knee arthroplasty: Definition and overview. Clin. Cases Min. Bone Metab..

[9] Carulli C., Matassi F., Nistri L., Civinini R., Innocenti M. (2012). Long-term survival of a flat-on-flat total condylar knee arthroplasty fixed with a hybrid cementing technique for tibial components. J. Long. Term. Eff. Med. Implant..

[10] Carulli C., Leggieri F., Rodà D., Matassi F., Civinini R., Innocenti M. (2024). Comparison of hypoallergenic knee arthroplasties in patients with metal hypersensitivity versus standard arthroplasties in non-hypersensitivity patients: A scoping review. J. Orthop..

[11] David M., Assil M., Yaniv Y., Yaron B. (2024). Comparing Complication Rates, Costs, and Length of Stay between Unicompartmental and Total Knee Arthroplasty: Insights from a Big Data Analysis Using the National Inpatient Sample Dataset. J. Clin. Med..

[12] Villeco J.P. (2012). Edema: A silent but important factor. J. Hand Ther..

[13] Río-González Á., Delgado-Pérez E., García-García E., González-Fernández L., García-Isidoro S., Cerezo-Téllez E. (2025). Physiotherapy Intervention in the Immediate Postoperative Phase of Lipedema Surgery—Observational Study. J. Clin. Med..

[14] Ramanujam V., Bessette J., Yeh J., Shah Y., Moazezi B., Kendall M.C. (2026). Advances in Peripheral Nerve Block Techniques and Clinical Strategies for Their Implementation Following Total Knee Arthroplasty: A Narrative Review. J. Clin. Med..

[15] Joo J., Kim M.S., Lee J., Koh H.J. (2025). What Is the Most Effective Strategy for Acute Postoperative Pain in Total Knee Arthroplasty—Retrospective Observational Study. J. Clin. Med..

[16] Coluzzi F., Di Martino A. (2025). “Pain Prehabilitation” in Major Joint Surgery: The Way Forward to Improve Outcomes and Prevent Pain Chronicity. J. Clin. Med..

[17] Lim J., Kim B. (2025). Effects of Resistance Training on Pain, Muscle Strength, and Function in Patients Undergoing Total Knee Arthroplasty: A Systematic Review and Meta-Analysis. J. Clin. Med..

[18] Brix B., Apich G., Roessler A., Ure C., Schmid-Zalaudek K., Hinghofer-Szalkay H., Goswami N. (2020). Fluid Shifts Induced by Physical Therapy in Lower Limb Lymphedema PatientsJ. Clin. Med..

[19] Rìo Gonzàlez A. (2020). Effects of Different Neck Manual Lymphatic Drainage Maneuvers on the Nervous, Cardiovascular, Respiratory and Musculoskeletal Systems in Healthy Students. J. Clin. Med..

[20] Kasseroller R.G. (1998). The Vodder School: The Vodder method. Cancer.

[21] Huang T.W., Tseng S.H., Lin C.C., Bai C.H., Chen C.S., Hung C.S., Wu C.H., Tam K.W. (2013). Effects of manual lymphatic drainage on breast cancer-related lymphedema. World J. Surg. Oncol..

[22] Földi E. (1998). The treatment of lymphedema. Cancer.

[23] Lu H., Shao Q., Li W., Li F., Xiong W., Li K., Feng W. (2024). Effects of manual lymphatic drainage on total knee replacement: A systematic review and meta-analysis of randomized controlled trials. BMC Musculoskelet. Disord..

[24] Migliorini F., Schäfer L., Bertini F.A., Memminger M.K., Simeone F., Giorgino R., Maffulli N. (2023). Level I of evidence does not support manual lymphatic drainage for total knee arthroplasty: A meta-analysis. Sci. Rep..

[25] Page M.J., McKenzie J.E., Bossuyt P.M., Boutron I., Hoffmann T.C., Mulrow C.D., Shamseer L., Tetzlaff J.M., Akl E.A., Brennan S.E. (2021). The PRISMA 2020 statement: An updated guideline for reporting systematic reviews. BMJ.

[26] Vergili Ö., Canbeylï I.D., Özsar B.K., Oktaş B., Keskin S. (2022). The effect of manual lymphatic drainage on postoperative recovery process following total knee arthroplasty. J. Med. Palliat. Care.

[27] Pichonnaz C., Bassin J.-P., Lécureux E., Christe G., Currat D., Aminian K., Jolles B.M. (2016). Effect of Manual Lymphatic Drainage after Total Knee Arthroplasty: A Randomized Controlled Trial. Arch. Phys. Med. Rehabil..

[28] Weber M., Oppermann J., Lummer C. (2025). Postoperative swelling: Influence of a negative pressure application in comparison to manual lymphatic drainage after total knee arthroplasty—A randomized controlled trial. Eur. J. Orthop. Surg. Traumatol..

[29] Wagner M., Wittlinger A., Auffarth A., Endstrasser F., Neururer S., Brunner A. (2024). Manual lymphatic drainage before and after total knee arthroplasty: A randomized controlled trial. J. Clin. Orthop. Trauma.

[30] Guney-Deniz H., Kinikli G.I., Aykar S., Sevinc C., Caglar O., Atilla B., Yuksel I. (2023). Manual lymphatic drainage and Kinesio taping applications reduce early-stage lower extremity edema and pain following total knee arthroplasty. Physiother. Theory Pract..

[31] Tornatore L., De Luca M.L., Ciccarello M., Benedetti M.G. (2020). Effects of combining manual lymphatic drainage and Kinesiotaping on pain, edema, and range of motion in patients with total knee replacement. Int. J. Rehabil. Res..

[32] Ebert J.R., Joss B., Jardine B., Wood D.J. (2013). Randomized trial investigating the efficacy of manual lymphatic drainage to improve early outcome after total knee arthroplasty. Arch. Phys. Med. Rehabil..

[33] Fujiura T., Nagasawa H., Wakabayashi H. (2020). Effect of manual lymph drainage for up to 10 days after total knee arthroplasty: A randomized controlled trial. Phys. Ther. Res..

[34] Gilchrist L., Levenhagen K., Davies C.C., Koehler L. (2024). Effectiveness of complete decongestive therapy for upper extremity breast cancer-related lymphedema: A review of systematic reviews. Med. Oncol..

[35] Ezzo J., Manheimer E., McNeely M.L., Howell D.M., Weiss R., I Johansson K., Bao T., Bily L., Tuppo C.M., Williams A.F. (2015). Manual lymphatic drainage for lymphedema following breast cancer treatment. Cochrane Database Syst. Rev..

[36] Thompson B., Gaitatzis K., De Jonge X.J., Blackwell R., Koelmeyer L.A. (2021). Manual lymphatic drainage treatment for lymphedema: A systematic review of the literature. J. Cancer Surviv..

