# Supplementary material for: Effectiveness of Manual Lymphatic Drainage After Total Knee Arthroplasty: A Systematic Review
Source: J Clin Med. 2026 Jul 16;15(14):5575. doi: 10.3390/jcm15145575 (PMC13413183; doi:10.3390/jcm15145575)
Supplement: Supplementary file 1 [file jcm-15-05575-s001.zip › jcm-4384708-PRISMA 2020 Checklist.pdf]

PRISMA 2020 Checklist

Page, M.J.; McKenzie, J.E.; Bossuyt, P.M.; Boutron, I.; Hoffmann, T.C.; Mulrow, C.D.; Shamseer, L.; Tetzlaff, J.M.; Akl, E.A.; Brennan, S.E.; et al. The PRISMA 2020 statement: an updated guideline for reporting systematic reviews BMJ 2021, 372, n71

Section	Item #	Checklist Item	Location in Manuscript
TITLE	1	Identify the report as a systematic review.	Title: "Effectiveness of Manual Lymphatic Drainage in the Treatment of Postoperative Edema after Knee Arthroplasty: A Systematic Review"
ABSTRACT	2	See the PRISMA 2020 for Abstracts checklist.	Abstract not separately provided; introduction, methods, results and conclusions are present in the manuscript body.
**INTRODUCTION**			
Rationale	3	Describe the rationale for the review in the context of existing knowledge.	Introduction, paragraph 4: rationale for reviewing MLD evidence in post-TKA rehabilitation described, noting that evidence remains "inconclusive".
Objectives	4	Provide an explicit statement of the objective(s) or question(s) the review addresses.	Introduction, final paragraph: "The present systematic review aimed to critically evaluate the available evidence from RCTs regarding the effectiveness of MLD on edema, pain, and ROM in the early postoperative period following TKA".
**METHODS**			
Eligibility criteria	5	Specify the inclusion and exclusion criteria for the review and how studies were grouped for the syntheses.	Materials and Methods, "Eligibility Criteria": PICO framework table (Population, Intervention, Comparison, Outcomes, Study design); exclusion criteria listed explicitly.
Information sources	6	Specify all databases, registers, websites, handsearching, reference lists, individuals/organizations contacted, and date of last search/consultation.	Materials and Methods, "Information Sources and Search Strategy": PubMed (MEDLINE), Embase, CINAHL, Google Scholar; search up to June 2025; reference lists manually screened.
Search strategy	7	Present the full search strategies for all databases and registers, including filters applied.	Materials and Methods, "Information Sources and Search Strategy": MeSH terms and free-text keywords described; full search strings referenced in Appendix A.
Selection process	8	Specify the methods used to decide whether a study met the inclusion criteria of the review, including how many reviewers screened each record and whether they worked independently, and if applicable, details of automation tools used in the process.	Materials and Methods, "Study Selection and Data Extraction": title/abstract screening followed by full-text assessment performed by a single reviewer; reference lists manually screened; study management via Mendeley.
Data collection process	9	Specify the methods used to collect data from reports, including how many reviewers collected data from each report, any processes for obtaining or confirming data from study investigators, and if applicable,	Materials and Methods, "Study Selection and Data Extraction": data extracted by a single reviewer; variables extracted listed explicitly.

Section	Item #	Checklist Item	Location in Manuscript
		details of automation tools used in the process.	
Data items	10	List and define all outcomes for which data were sought. Specify whether all results that were compatible with each outcome domain in each study were sought, and if so, the methods used for finding these.	Materials and Methods, "Eligibility Criteria" (PICO table) and "Outcomes and Follow-up": primary outcome = edema (limb circumference, volumetry, BIS); secondary = pain (VAS/NRS) and ROM.
Study risk of bias assessment	11	Specify the methods used to assess risk of bias in the included studies, including details of the tool(s) used, how many reviewers assessed each study and whether they worked independently, and if applicable, details of automation tools used in the process.	Materials and Methods, "Methodological Quality Assessment": Cochrane Risk of Bias 2 (RoB 2) tool; five domains examined. Single reviewer.
Effect measures	12	Specify for each outcome the effect measure(s) (e.g. risk ratio, mean difference) used in the synthesis or presentation of results.	Materials and Methods, "Statistical Analysis": standardized mean differences (SMD) used for meta-analysis; random-effects model (REML); Knapp-Hartung adjustments; I^2 for heterogeneity.
Synthesis methods	13	Describe the processes used to decide which studies were eligible for each synthesis, how data were prepared for presentation in syntheses, any processes used to standardize or convert data to a common effect measure, and statistical methods used to synthesize data.	Materials and Methods, "Statistical Analysis": random-effects model (REML estimator) for outcomes with ≥2 comparable studies; R/RStudio used; outcomes not amenable to meta-analysis reported narratively.
Reporting bias assessment	14	Describe any methods used to assess risk of bias due to missing results in a synthesis (arising, for example, from selective reporting in studies and non-reporting of some outcomes).	Not explicitly reported. Narrative discussion in Discussion acknowledges methodological limitations of included studies (underpowered studies, short follow-up). Limitation to note for revision.
Certainty assessment	15	Describe any methods used to assess certainty (or confidence) in the body of evidence for an outcome.	Not explicitly reported (e.g., GRADE not mentioned). RoB 2 assessment is used to evaluate methodological quality. Limitation to note for revision.
**RESULTS**			
Study selection	16a	Describe the results of the search and selection process, including reasons for exclusion at each stage of selection.	Results, paragraph 1: 550 records identified; 47 retrieved after title/abstract screening; 26 duplicates removed; 13 excluded after full-text evaluation; 8 RCTs included. PRISMA flow diagram referenced as Figure 1.
Study selection	16b	Cite the study identification tools (e.g. PRISMA flow diagram) used.	Results: "Eight RCTs were ultimately included in the systematic review (Figure 1)". Flow diagram present as Figure 1.

Section	Item #	Checklist Item	Location in Manuscript
Study characteristics	17	Cite each included study and present its characteristics.	Results, Tables 2 and 3: author, year, country, sample size, mean age, interventions, comparators, session details provided for all 8 RCTs.
Risk of bias in studies	18	Present assessments of risk of bias for each included study.	Results, "Risk of Bias": RoB 2 assessment described; blinding limitations noted; randomization and outcome measurement generally adequate; Figure 2 referenced.
Results of individual studies	19	For all outcomes, present, for each study, either (a) summary statistics for each group (where appropriate) or (b) effect estimate and precision for each outcome.	Results, "Narrative Synthesis of Individual Study Results": individual study results summarized for all 8 RCTs including key outcome data for edema, pain, and ROM at each time point.
Results of syntheses	20a	For each synthesis, briefly summarise the characteristics and risk of bias among contributing studies.	Results, "Meta-Analysis Results": pooled SMD reported for edema (−0.27; I^2=78.1%) and AROM (0.15; I^2=0.0%); heterogeneity noted.
Results of syntheses	20b	Present results of all statistical syntheses conducted. If a meta-analysis was done, present for each the summary estimate and its precision, the amount of heterogeneity, and the CI/CrI of the heterogeneity measure.	Results, "Meta-Analysis Results": SMD, 95% CI, I^2, and p-values reported for both meta-analyses (edema and AROM). Forest plots referenced (Plot 1, Plot 2).
Results of syntheses	20c	If comparing groups, describe the direction of the effect for the favoured group.	Results: both pooled SMDs reported as non-significant; direction of effect described (negative for edema, positive for AROM). No group consistently favoured.
Results of syntheses	20d	Report results of any investigations of possible causes of heterogeneity among study results.	Results: I^2 reported for both meta-analyses; high heterogeneity in edema analysis (I^2=78.1%) noted. No formal subgroup analysis or meta-regression reported — limitation to note for revision.
Reporting biases	21	Present assessments of risk of bias due to missing results (arising from selective reporting in studies and non-reporting of results).	Not formally assessed (no funnel plot or Egger test reported). Acknowledged implicitly through the RoB 2 domain on "selection of reported results". Limitation to address.
Certainty of evidence	22	Present assessments of certainty (or confidence) in the body of evidence for an outcome.	Not formally assessed with GRADE or equivalent tool. Study quality discussed narratively. Limitation to note for revision.
**DISCUSSION**			
Discussion	23a	Provide a general interpretation of the results in the context of other evidence.	Discussion, paragraph 1–3: findings contextualized against Gilchrist 2024, Ezzo 2015, Thompson 2021; differences between post-TKA edema and lymphedema models discussed.
Discussion	23b	Discuss any limitations of the evidence included in the review.	Discussion, paragraph 4: limitations include small sample sizes, short follow-up, reliance on circumference measurement, and lack of MLD protocol standardization.
Discussion	23c	Discuss any limitations of the review processes used.	Discussion, paragraph 4: single-reviewer selection and extraction noted implicitly; limitations related to measurement tools and follow-up discussed.
**OTHER INFORMATION**			

Section	Item #	Checklist Item	Location in Manuscript
Registration and protocol	24a	Provide registration information for the review, including register name and registration number, or state that the review was not registered.	Not reported in the manuscript. Authors should verify whether registration (e.g., PROSPERO) was performed and report accordingly.
Registration and protocol	24b	Indicate where the review protocol can be accessed, or state that a protocol was not prepared.	Not reported. If no protocol exists, this should be explicitly stated.
Registration and protocol	24c	Describe and explain any amendments to information provided at registration or in the protocol.	Not applicable if not registered.
Support	25	Describe sources of financial or other support for the review, and the role of the funders or sponsors in the review.	Manuscript states: "This research received no external funding."
Competing interests	26	Declare any competing interests of review authors.	Manuscript states: "The authors declare no conflicts of interest."
Availability of data, code and other materials	27	Report which of the following are publicly available and where they can be found: template data collection forms; data extracted from included studies; data used for all analyses; analytic code; any other materials used in the review.	Manuscript states: "No new data were created or analysed in this study. Data sharing is not applicable."

Note: Items marked as 'Not reported' or 'Limitation to note for revision' indicate areas where the manuscript may benefit from additional clarification before final submission.
