# Supplementary material for: Effectiveness of Manual Lymphatic Drainage After Total Knee Arthroplasty: A Systematic Review
Source: J Clin Med. 2026 Jul 16;15(14):5575. doi: 10.3390/jcm15145575 (PMC13413183; doi:10.3390/jcm15145575)
Supplement: Supplementary file 1 [file jcm-15-05575-s001.zip › jcm-4384708-supplementary Figure S1.pdf]

Study	D1 Randomization	D2 Deviations	D3 Missing data	D4 Outcome measurement	D5 Selective reporting
Weber	Low	Some concerns	Low	Low	Some concerns
Wagner	Low	Some concerns	High	Low	Some concerns
Guney-Deniz	Some concerns	Some concerns	High	High	Some concerns
Tornatore	Some concerns	Some concerns	Low	High	Some concerns
Pichonnaz	Low	Some concerns	Low	Low	Low
Ebert	Low	Some concerns	Low	Low	Low
Fujiura	Some concerns	Some concerns	Low	High	Some concerns
Vergili	High	Some concerns	High	High	Some concerns

Figure S1. Risk of bias summary for included studies.
