# Supplementary material for: Effectiveness of Manual Lymphatic Drainage After Total Knee Arthroplasty: A Systematic Review
Source: J Clin Med. 2026 Jul 16;15(14):5575. doi: 10.3390/jcm15145575 (PMC13413183; doi:10.3390/jcm15145575)
Supplement: Supplementary file 1 [file jcm-15-05575-s001.zip › jcm-4384708-supplementary File S1.pdf]

Search strings:

PUBMED

("Arthroplasty, Replacement, Knee"[Mesh] OR "Knee Prosthesis"[Mesh] OR ("Joint Prosthesis"[Mesh] AND knee[tiab]) OR ("Prosthesis Implantation"[Mesh] AND knee[tiab]) OR tkr OR (knee* AND arthroplast*) OR "knee arthroplasty*" OR (knee* AND replacement*) OR "knee replacement*" OR (total* AND knee* AND (replacement* OR arthroplast*)) OR "total knee replacement*" OR "total knee arthroplasty*" OR ((knee replacement) AND (arthroplast*)) OR "knee prosthe*" OR "knee endoprosthe*" OR ("partial knee" AND (replacement* OR arthroplast*)) OR (unicompartmental knee* AND (replacement* OR arthroplast*)) OR ("unicondylar knee" AND (replacement* OR arthroplast*)) OR (("knee joint"[MeSH Terms] OR (knee* AND joint*) OR "knee joint*" OR knee*) AND (replaced OR replacement* OR "replantation"[MeSH Terms] OR replantation OR arthroplast* OR prosthe*)) OR "Knee Replacement Arthroplasties" OR "Knee Replacement Arthroplasty" OR "Knee Arthroplasty" OR "Knee Replacement" OR "Unicompartmental Knee Arthroplasty" OR "Unicompartmental Knee" OR "Unicompartmental" OR "Unicompartmental Knee Replacement" OR "Partial Knee Replacement" OR "Unicondylar Knee Replacement" OR "Unicondylar Knee Arthroplasty" OR "Unicondylar Knee" OR "Partial Knee Arthroplasty" OR (knee* AND (prosthes* OR endoprosthes*)))

AND

("Edema"[Mesh] OR "Lymphedema"[Mesh] OR "Seroma"[Mesh] OR edema* OR oedema* OR anasarca OR hydrops OR dropsy OR ((limb OR surgical OR postoperative OR knee OR peripheral OR chronic OR localized) AND (swelling OR effusion OR edema* OR oedema*)) OR ((lymphatic*) AND (insufficiency* OR dysfunction OR impairment*)) OR "soft tissue swelling" OR "secondary lymphedema" OR ((edematous OR oedematous) AND (tissue OR limb)))

AND

("Manual Lymphatic Drainage"[Mesh] OR ("Lymphedema"[Mesh] AND ("Therapy"[Subheading] OR rehabilitation)) OR (lymph* AND (drainage* OR massage* OR manual*)) OR (lymphatic* AND (drainage* OR massage* OR manual*)) OR (manual* AND (lymph* OR lymphatic* OR drainage* OR massage*)) OR "Vodder" [tiab] OR “Foldi” [tiab] OR "Exercise Therapy"[Mesh] OR "Bandages"[Mesh] OR "Skin Care"[Mesh] OR "Self Care"[Mesh] OR "Physical Therapy Modalities"[Mesh] OR "complex decongestive therapy" OR "CDT" OR "Decongestive Lymphatic Therapy" OR "complete decongestive therapy" OR "decongestive physiotherapy" OR "combined decongestive therapy" OR "decongestive lymphatic therapy" OR ((complex*) AND (decongestive therap*) OR (decongestive physiotherap*) OR (lymphatic therap*)) OR "comprehensive decongestive therapy" OR CDT OR CDPT OR (compression AND (bandage* OR therap*)) OR ((multilayer* AND short-stretch) AND (bandaging*)) OR "intermittent pneumatic compression device" OR "compression devices" OR "intermittent pneumatic" OR "pneumatic intermittent impulse device" OR "pneumatic compression stockings" OR "compression stocking" OR ((pneumatic*) AND (compression* OR hose*)) OR IPC OR ((compression*) AND (wrap*)) OR ((elastic*) AND (compression* OR bandag*)) OR bandag* OR stocking* OR wrap* OR ((therapeutic* OR resistance OR activ* OR functional* OR rehabilitation) AND (exercis*)) OR "home-based therapy" OR "home program" OR "self-management")

EMBASE

(('knee replacement'/exp OR 'knee replacement' OR 'knee prosthesis'/exp OR 'knee prosthesis' OR (('joint prosthesis'/exp OR 'joint prosthesis') AND ('knee'/exp OR 'knee')) OR 'knee'/exp OR 'knee') AND ('arthroplasty'/exp OR 'arthroplasty') OR (knee:ti,ab AND replacement:ti,ab) OR 'knee replacement':ti,ab OR 'knee arthroplasty':ti,ab OR 'unicompartmental knee prosthesis'/exp OR 'unicompartmental knee prosthesis') AND ('edema'/exp OR 'edema' OR 'lymphedema'/exp OR 'lymphedema' OR 'seroma'/exp OR 'seroma' OR 'anasarca'/exp OR 'anasarca' OR 'ascites'/exp OR 'ascites' OR (('limb'/exp OR 'limb' OR surgical:ti,ab OR postoperative:ti,ab OR perypheral:ti,ab OR chronic:ti,ab OR localized:ti,ab) AND ('joint swelling'/exp OR 'joint swelling' OR swelling:ti,ab OR 'effusion'/exp OR 'effusion' OR edema:ti,ab)) OR 'soft tissue swelling':ti,ab OR

'secondary lymphedema':ti,ab) AND ('manual lymphatic drainage'/exp OR 'manual lymphatic drainage' OR (('lymphedema'/exp OR 'lymphedema') AND ('therapy'/exp OR 'therapy' OR 'massage'/exp OR 'massage' OR 'drainage':ti,ab OR bandages:ti,ab)) OR vodder:ti,ab OR foldi:ti,ab OR 'lymphatic therapy':ti,ab OR 'decongestive therapy':ti,ab OR 'decongestive physiotherapy':ti,ab OR 'intermittent pneumatic compression device'/exp OR 'intermittent pneumatic compression device' OR cdpt:ti,ab OR cdt:ti,ab OR 'pneumatic intermittent impulse device':ti,ab OR 'compression stocking'/exp OR 'compression stocking' OR (pneumatic:ti,ab AND (hose:ti,ab OR compression:ti,ab)) OR ((wrap:ti,ab OR elastic:ti,ab) AND (bandage*:ti,ab OR stocking*:ti,ab)))

CINHAL

((MH "Edema+" OR MH "Lymphedema+" OR XB (edema* OR oedema* OR anasarca OR hydrops* OR dropsy OR ((limb OR surgical OR postoperative OR knee OR peripheral OR chronic OR localized) AND (swelling OR effusion OR edema* OR oedema*)) OR ((lymphatic*) AND (insufficiency* OR dysfunction OR impairment*)) OR "soft tissue swelling" OR "secondary lymphedema" OR ((edematous OR oedematous) AND (tissue OR limb)))) AND (MM "Arthroplasty, Replacement, Knee+" OR ((MH "Knee" AND MH "Joint Prosthesis+")) OR XB ((knee* AND arthroplast*) OR "knee arthroplasty*" OR (knee* AND replacement*) OR "knee replacement*" OR (total* AND knee* AND (replacement* OR arthroplast*)) OR "total knee replacement*" OR "total knee arthroplasty*" OR "knee prosthe*" OR "knee endoprosthe*" OR ("partial knee" AND (replacement* OR arthroplast*)) OR ("unicompartmental knee*" AND (replacement* OR arthroplast*)) OR ("unicondylar knee" AND (replacement* OR arthroplast*)) OR "Knee Replacement Arthroplasties" OR "Knee Replacement Arthroplasty" OR "Knee Arthroplasty" OR "Knee Replacement" OR "Unicompartmental Knee Arthroplasty" OR "Unicompartmental Knee" OR "Unicompartmental" OR "Unicompartmental Knee Replacement" OR "Partial Knee Replacement" OR "Unicondylar Knee Replacement" OR "Unicondylar Knee Arthroplasty" OR "Unicondylar Knee" OR "Partial Knee Arthroplasty")) AND ((MH "Manual Lymphatic Drainage" OR XB ((lymph* AND (drainage* OR massage* OR manual*)) OR (lymphatic* AND (drainage* OR massage* OR manual*)) OR (manual* AND (lymph* OR lymphatic* OR drainage* OR massage*)) OR "Vodder" OR “Foldi” OR "complex decongestive therapy" OR "CDT" OR "Decongestive Lymphatic Therapy" OR "complete decongestive therapy" OR "decongestive physiotherapy" OR "combined decongestive therapy" OR "decongestive lymphatic therapy" OR ((complex*) AND (decongestive therap*) OR (decongestive physiotherap*) OR (lymphatic therap*)) OR "comprehensive decongestive therapy" OR CDT OR CDPT OR (compression AND (bandage* OR therap*)) OR ((multilayer* AND short-stretch) AND (bandaging*)) OR "intermittent pneumatic compression device" OR "compression devices" OR "intermittent pneumatic" OR "pneumatic intermittent impulse device" OR "pneumatic compression stockings" OR "compression stocking" OR ((pneumatic*) AND (compression* OR hose*)) OR IPC OR ((compression*) AND (wrap*)) OR ((elastic*) AND (compression* OR bandag*)) OR bandag* OR stocking* OR wrap* OR ((therapeutic* OR resistance OR activ* OR functional* OR rehabilitation) AND (exercis*)) OR "home-based therapy" OR "home program*" OR "self-management")))

GOOGLE SCHOLAR

("knee replacement" OR "knee arthroplasty" OR "total knee replacement" OR "partial knee replacement" OR "unicompartmental knee arthroplasty" OR "unicondylar knee replacement" OR "knee prosthesis")

AND

("lymphedema" OR "secondary lymphedema" OR "edema" OR "postoperative edema" OR "post-surgical swelling" OR "soft tissue swelling")

AND

("manual lymph drainage" OR "complex decongestive therapy" OR "complete decongestive therapy" OR "decongestive physiotherapy" OR "CDT" OR "lymphatic drainage" OR "compression therapy" OR "compression bandaging" OR "pneumatic compression" OR "intermittent pneumatic compression" OR "compression stockings" OR "compression devices" OR "physical therapy" OR "rehabilitation" OR "exercise therapy" OR "bandaging" OR "self-management" OR "home program" OR "Vodder method" OR "Foldi method")
